# Supplementary material for: A geometric shape regularity effect in the human brain
Source: eLife. 2026 Jan 12;14:RP106464. doi: 10.7554/eLife.106464 (PMC12795501; doi:10.7554/eLife.106464)
Supplement: Figure 1—video 1—source data 1. [file elife-106464-fig1.docx]

00:00
 In this work, we are interested in understanding the mechanisms that underlie the representation of geometric shapes in the human brain. We designed shapes matched for visual properties such as area or side length, but that vary in number of geometric features such as right angles, parallel sides, sides of equal length, or angles of equal measure. In the first panel, we show the 11 shapes that we will use across the experiments featured here, ranked by the complexity of their mental representations as measured in previous work.

00:32
 We start with a simple behavior task to estimate an empirical distance measure between our geometric shapes. In a display of nine shapes with some jitter in scale and rotation, participants had to find the intruder by clicking on it. In panel B, we display two trials featuring the square and the rhombus. In both examples, the intruder is in the bottom left position, but of course in the experiment, the position was randomized. Participants took two trials for each pair of shapes. The order of the trials was randomized.

01:01
 We aggregate response times and error rates to measure an empirical distance between shapes. On the heat map left of panel D, you can see how dissimilar any two shapes are. For example, the square and the rectangle are quite dissimilar from all of the other shapes, but quite similar from one another. We can visualize these distances in a 2D space. In panel C, you can see the multidimensional scaling projection.

01:27
 The first dimension already separates shapes by intuitive complexity, as it puts the square and the rectangles all the way to the right, the rhombi in the center, and the other shapes on the left. The arrow shows the projection on these dimensions of the number of geometric features of each shape. It is significantly different from zero in both dimensions. We can model the dissimilarity matrix with different theories of shape complexity. In panel D, we show two.

01:55
 In orange, a geometric feature model, which counts the number of exact geometric features, such as right angle, that are different between shapes. In purple, a model based on a convolutional neural network encoding. The one displayed here looks at the distance in the penultimate layer of coordinate S after it was trained for object recognition.

02:17
 For each participant, we display the regression coefficient of its dissimilarity matrix with both models. As you can see in panel E, both models are significant predictors of the behavior, and the geometric feature model is about three times as big as the CNN one. In the rest of the article, we will investigate the neural basis of this finding. If you want more details about the models, we reference an article from 2021 that details them.

02:43
 We also have supplementary figures that display other neural networks, other layers, or how robust this result is to the exact choice of shapes. And in particular, if you focus on shapes that do not have a name in English.
