# Supplementary material for: A geometric shape regularity effect in the human brain
Source: eLife. 2026 Jan 12;14:RP106464. doi: 10.7554/eLife.106464 (PMC12795501; doi:10.7554/eLife.106464)
Supplement: Figure 2—video 1—source data 1. [file elife-106464-fig2.docx]

00:01
 To understand the neural basis of our findings, we turn to fMRI and we first report results from a visual localizer in adults and six-year-old children. As participants are lying in the scanner, they see blocks of images that belong to the same visual category. We used eight visual categories, shown in panel A. To maintain attention, occasionally the image displayed was a star that participants had to report by pressing a button, as shown in panel B.

00:30
 We report in panel C the contrast between a single shape and the other visual categories with a single thing on screen, faces, tools, and houses. In adults, they solicit two notable features, a bilateral underactivation of the ventral pathways and two overactivations in the right hemisphere, in the anterior intraperidal sulcus and in the posterior inferior temporal gyrus. In children, we find a very similar bilateral underactivation.

00:57
 And we also find the right anterior IPS activation, as well as its left counterpart. We do not find an overactivation in the posterior ITG. Specifically testing the children's left IPS in adults reveals a significant overactivation too. And testing the adults' right ITG in children reveals a significant overactivation as well. To see how similar the statistical maps are, you can find the uncorrected statistical maps in supplementary figure.

01:26
 you will also find the statistical maps associated to other contrasts. Finally, for each of the five regions of interest, two underactivations and three overactivations, we display the average coefficients associated to each visual category together with a standard error across subjects in both populations. From this simple analysis with passive perception, we can already see that geometric shapes activate a cortical network that goes beyond object recognition.

01:55
 and has overlap with areas found in tasks with mathematical content. In fact, in adults in panel D, you can see that the left IPS is even more activated by our number stimuli. In supplementary material, we carefully parcellate the ventral pathway and study its response to our shapes.
