# Supplementary material for: A geometric shape regularity effect in the human brain
Source: eLife. 2026 Jan 12;14:RP106464. doi: 10.7554/eLife.106464 (PMC12795501; doi:10.7554/eLife.106464)
Supplement: Figure 3—video 1—source data 1. [file elife-106464-fig3.docx]

00:00
 The previous results broadly identified regions that are sensitive to geometric shapes, but we would like to understand how the differences between shapes are represented. We therefore turned to a simple intruder task inside the fMRI. Participants saw six shapes identical up to rotation and scaling, except for one intruder constructed by moving the bottom right corner a little bit. Participants had to say whether the intruder was on the left or the right by pressing a button.

00:26
 For time constraints, we only used six out of our 11 shapes. In panel B, you can see that both populations perform better than chance. If we correlate their behavior inside the scanner to behavior from a bigger cohort tested online, we see that they perform very similarly. And we see a gradient of complexity whereby simple shapes elicit fewer mistakes than more complex shapes. We would like to understand the neural basis of that gradient.

00:55
 First, we can look at which areas have a signal that scales with shape complexity. This reveals a broad bilateral network in adults. The inset scatter plots with regressions show the only two areas identified in adults that are close to significance in children.

01:11
 Because of the task design, this cortical network includes areas that encode shapes, but also areas involved in the decision process. This is therefore confounded by task difficulty and attention. This is why we then turned to a representational similarity analysis. For each voxel and its surrounding sphere, we estimated the distance between all pairs of shapes. This provides us with a dissimilarity matrix, just like we had for behavior, which we can compare to our two models.

01:41
 The plot in panel D shows the significant clusters associated with each model, orange for geometry, purple for CNN, and green when they overlap. You can see that visual areas, as well as the right premotor cortex and the anterior cingulate cortex, are activated by both models, probably reflecting pooling of the information for decision-making, while the dorsal pathway, together with the frontal pole, appear to encode geometric features only.
