# Supplementary material for: A geometric shape regularity effect in the human brain
Source: eLife. 2026 Jan 12;14:RP106464. doi: 10.7554/eLife.106464 (PMC12795501; doi:10.7554/eLife.106464)
Supplement: Figure 4—video 1—source data 1. [file elife-106464-fig4.docx]

00:00
The previous ephemera experiment was confounded by attention and task difficulty. To prevent this, and to understand the spatio-temporal dynamic of geometric shape processing, we turned to MEG in a very simple task, a passive oddball paradigm. Shapes were presented in blocks of 30 of the same kind, with random rotation and scaling. Within these 30 shapes, we inserted four deviants.

00:23
 For example, the experiment might start with presenting 30 squares identical up to rotation and scaling, except for a few deviants. While we recorded data, participants were simply asked to pay attention to the shape on screen. First, we find in the neural data that despite the absence of a task, participants detected intruders. We could successfully train a classifier on the sensor data that predicts better than chance whether a given trial is an intruder or not.

00:53
 In panel D, on the left, we show the performance over time of this decoder when applied to each shape separately. As you can see, decoding intruders within squares is much easier than within irregular shapes. In the second plot, you can see the correlation between the decoder's accuracy on each shape and that shape's complexity. There is a big significant cluster from about 150 milliseconds onward. The scatter plot to the right displays the average correlation inside this cluster.

01:24
 This could be because the decoder finds a direction in sensor space that is strongly tuned for intruders with its squares and performance degrade for other shapes. To ensure that this is not the case, we separately trained 11 decoders, one for each shape, displayed in panel C. Even though all of them perform better than chance, we find again that intruders are easier to decode with irregular than irregular shapes, although everything is noisier as the decoders are trained on less data.

01:52
 To better understand the neural dynamic of shape processing, we rely on representational similarity analysis. At each time point, we estimate the distance between any two shapes in sensor activity. Then, we model this distance with our two models of shape representation. We find that both are significant, but at different times. The CNN peaks first around 90 milliseconds, while the geometric model peaks around 230 milliseconds.

02:19
 At the bottom of panel D, you can see the empirical dissimilarity matrices at the peak of their correlation. In this video, I added as an additional insert our models for reference. Finally, we zoom in on the timings identified in the sensor space analysis. Then,

02:36
 we project sensor activity back onto cortical sources and perform our analysis again, but now using a searchlight scanning across the whole brain. As you can see in panel E, the neural network model is strongest in visual areas, while the geometric model activates more anterior parts of the visual pathways, as well as dorsal frontal and medial frontal areas.

02:55
 You can find a supplementary file containing a movie with the entire spatiotemporal dynamic of this analysis across the brain. From this, we conclude that shapes are first processed with the typical visual pathways, but that even in the absence of a task, they are then mentally encoded with their exact symbolic properties in a broad network that includes the frontal cortex. Thank you for listening.
